# Creating an Internal Environment of Cognitive and Psycho-Emotional Well-Being through an External Movement-Based Environment: An Overview of Quadrato Motor Training

**DOI:** 10.3390/ijerph16122160

**Published:** 2019-06-18

**Authors:** Antonio De Fano, Rotem Leshem, Tal Dotan Ben-Soussan

**Affiliations:** 1 Research Institute for Neuroscience, Education and Didactics, Patrizio Paoletti Foundation, 06081 Assisi, Italy; a.defano@fondazionepatriziopaoletti.org; 2 Department of Criminology, Bar-Ilan Univesity, Ramat-Gan 5290002, Israel; rotem.leshem@biu.ac.il

**Keywords:** physical activity, Mindful Movement, cognition, emotion, built environment, social environment

## Abstract

In this overview, we discuss the internal and external environmental factors associated with cognitive and psycho-emotional well-being in the context of physical activity and Mindful Movement. Our key argument is that improved cognitive and emotional functions associated with mental well-being can be achieved by an external, Mindful Movement-based environment training called Quadrato Motor Training (QMT). QMT is a structured sensorimotor training program aimed at improving coordination, attention, and emotional well-being through behavioral, electrophysiological, neuroanatomical, and molecular changes. In accordance with this argument, we first describe the general neurobiological mechanisms underpinning emotional states and emotion regulation. Next, we review the relationships between QMT, positive emotional state, and increased emotion regulation, and discuss the neurobiological mechanisms underlying these relationships. We consider the relationships between motion, emotion, and cognition, and highlight the need for integrated training paradigms involving these three trajectories. Such training paradigms provide cognitively engaging exercises to improve emotion regulation, which in turn affects adaptive behaviors. Finally, we address the broader implications of improving cognitive and emotional functioning through Mindful Movement training for environmental research and public health.

## 1. Introduction

Cognitive and psycho-emotional well-being are associated with better physical and social health. In contrast, decreased cognitive and psycho-emotional functions are related to mental health concerns, such as anxiety and depression [1,2]. Cognition and emotion work together, jointly informing our impressions of situations and influencing social behavior. Together, they contribute to executive functions (EFs), which are involved in higher cognitive functions such as emotion regulation, attention, decision-making, creativity, and learning [3]. When well-established, EFs strengthen health and well-being [4].

In recent decades, neuroscientists have become increasingly interested in how the human brain modifies its structural and functional organization throughout the lifespan as a result of various external and internal determinants [5,6]. This phenomenon of neural adaptation and change is known as neuroplasticity [7,8]. It provides a scientific basis for developing, studying, and adopting practical interventions that promote health and well-being across the lifespan in both healthy and clinical populations.

In the current overview, we will discuss the internal and external environmental factors associated with cognitive and psycho-emotional well-being. Then, we will turn to research on physical activity and its influence on cognitive and emotional improvements, focusing on Quadrato Motor Training. We will conclude with the potential implications of this specific Mindful Movement practice for environmental research and public health.

### 1.1. Neuro-Bio-Sociological Environments are Substrates for Cognitive and Psycho-Emotional Well-Being

The concept of the *environment* can be defined in various ways. Here, environment refers to both internal and external settings, as follows: the internal environment encompasses intrinsic determinants, namely, the person’s neurological and physiological mechanisms, and the external environment comprises extrinsic determinants, including life events and experiences. Internal and external environments do not act independently on the individual, but rather work together to shape thoughts, feelings, and behaviors.

Within the neuro-bio-sociological framework, genes are the building blocks of cells, and interactions between cells eventually give rise to behaviors [9,10]. Genes play an important role in shaping behavior by encoding molecular products that build and govern the functioning of the brain, which in turn governs behavior [11]. Of the many different pathways by which genes influence the neural environment, one involves determination of the number and characteristics of neurons and the nature of the connections within and between brain regions. Another way in which genes affect behavior is by regulating neurotransmitters and receptors in the brain [11,12]. Environment and experience also act on the brain and affect behavior. For instance, adversities and negative life events may interfere with physiological developmental processes in the brain, leading to altered neural circuits that have been associated with behavioral phenotypes such as delinquency, physical aggression, depression, and anxiety [13,14]. Meanwhile, positive life experiences and enriched environments can nurture healthy brain function and behaviors [13,14]. Human mental states and behavioral outcomes are thus a reflection of environmental and genetic factors that impact the brain’s ability to adapt to changing environmental demands [9,10]. In other words, subjective well-being stems from bidirectional phenotypic adaptation to internal and external environments [14].

The flexibility of neural programing during critical periods seems to be a significant mediator of long-lasting effects on behavior [15,16]. Cognitive and emotional developments coincide with developmental changes in the brain. From birth, the brain rapidly creates connections between neurons that form our habits, thoughts, consciousness, memories, and mind [17,18]. Massive biological changes affect the gross morphology of the brain, including regressive processes (e.g., synaptic pruning), in which unused information is eliminated, and progressive processes (e.g., myelination), which increase the speed at which information travels between nerve cells [19,20,21]. Furthermore, in accordance with Hebbian theory [22], one of the most effective ways to create a more efficient brain and more focal recruitment of different brain areas, is to strengthen the synapses through repeated experiences and learning (for more details, see [23,24]). That is, learning results in more consolidation of neuronal activity and brain activity becomes more efficient, thus, every experience excites some neural circuits and leaves others unaffected. Increases in the efficacy of synaptic connections, including the connections between higher-order association areas in the frontal lobes, strengthen the ability to exert cognitive and emotional control [19,25], and are thought to support improvements in executive abilities such as response inhibition [26], strategic planning [27], and impulse regulation [28].

Overall, enhancing synaptic and neuronal activity at afferent locations throughout the brain affects the gross morphology of the brain and plays a particularly important role in the interaction between cognitive and emotional processes and their effects on behavior.

Support for interrelated cognitive-emotional processes can be found in studies indicating that executed, goal-directed behavioral responses require interactions and coordination between cognitive and emotional neural circuitries [19,29]. Specifically, different forms of cognitive and emotional processes (e.g., attentional control, emotion regulation) are products of the reciprocal interactions between frontal-subcortical circuits (FSCs) and limbic structures, such as the amygdala [30]. In addition, the prefrontal cortex (PFC) is often associated with changes in behavior and cognition that fall within the broader area of EFs, which play an important role in social and emotional wellness [4].

As detailed below, there is reason to believe that extrinsic determinants, such as physical activity (PA), contribute to the enhancement of interconnections between FSC networks and related EFs, which in turn increase cognitive and psycho-emotional well-being. We suggest that the execution of specifically structured PA practices can lead to integrative and balanced communication between cognition- and emotion-related association areas in the brain, helping to monitor, modify, and ultimately strengthen cognitive control processes [29]. Within this context, the current review is focused on a specific category of PA, called *Mindful Movement (MM)*.

### 1.2. The Impact of An External Movement-Based Environment on the Internal Environment

Of the interventions believed to enable neuroplasticity, PA is one of the most studied [31,32]. By building an external movement-based environment, it is possible to elicit positive neural changes throughout the lifespan. Through this process, PA reduces brain-related physiological and functional decline due to aging, and could potentially be used to improve several brain-related clinical conditions, including neurodegenerative diseases (e.g., dementia), psychiatric disorders (e.g., depression [33]), and neurodevelopmental disorders (e.g., autism and dyslexia) [32,34]. Future studies should be conducted in order to examine this important issue. Although the specific mechanisms underlying PA-induced neuroplasticity have not yet been elucidated, there are currently several hypotheses that attempt to explain this phenomenon. According to the neurotrophic hypothesis, the increased release of neurotrophic factors, such as brain-derived neurotrophic factor (BDNF) and nerve growth factor (NGF), is crucial [31,32,35], eliciting structural and functional brain changes like neurogenesis, synaptogenesis, neuronal survival and growth [36,37]. For additional details see [38]. Another hypothesized mechanism is related to electrophysiological changes. Most of the studies in this research field employed the event-related potential (ERP) components, such as the P3 component, error-related negativity (ERN), and contingent negative variation (CNV) [39]. Given the high temporal resolution (i.e., milliseconds), ERPs allow researchers to measure cognitive processes providing information regarding the mechanisms underlying cognitive functioning and the PA-induced effects [32]. Another important measure which is associated with PA-induced changes is alpha and theta enhancement, particularly in the frontal cortex [40]. However, as will be show in the next sections, studies conducted on a specific form of PA (called Quadrato Motor Training) suggest that also long-range synchronization between the activities of distinct neuronal populations (i.e., functional connectivity) involved in cognitive control, such as fronto-parietal and fronto-temporal networks, as another possible mechanism underlying the PA-induced neuroplasticity [29]. At the behavioral level, these changes have been associated with improvements in cognitive and psycho-emotional functioning [31,32,35,41], which are believed to be important aspects of health and well-being.

The neuroplastic effects of PA change according to its quantitative and qualitative features [42]. Historically, neuroscientific research in this area was focused on aerobic exercise and its metabolic demands, related fitness, and dose-response relations. As recently asserted by Pesce and colleagues [41,43], this trend was part of the perspective that exercise could be used as a medical tool to counteract diseases, such as obesity, cardiovascular diseases, and diabetes, as embodied in the assertion “Exercise is Medicine” [44]. In accordance, early studies on the effects of PA on the brain focused on understanding how and why aerobic exercise counteracts age-related decline in brain structure and functioning [43]. For example, several studies showed that aerobic exercise, such as jogging, running, cycling, and swimming improved neuroplasticity mainly in PFC-related regions and enhanced various cognitive abilities among children and older adults. However, these findings are in stark contrast with those reported by several other studies, which failed to show facilitative effects of aerobic exercise on cognition or selective improvements in a subset of cognitive functions, such as EFs e.g., [45,46].

**Figure 1:**
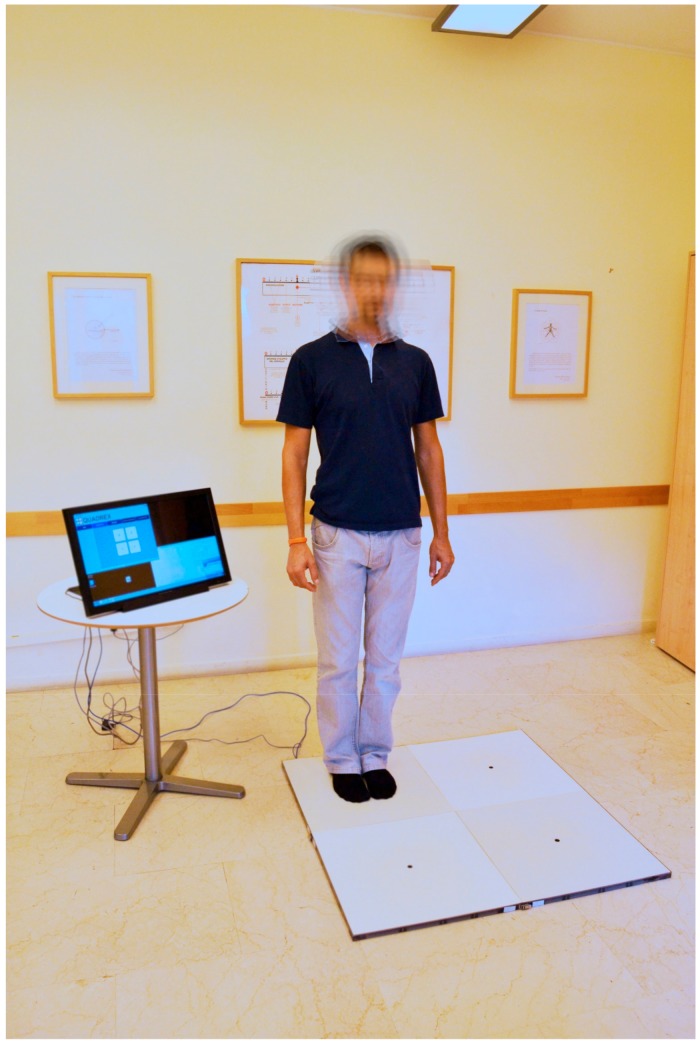
Quadrato Motor Training (QMT). A participant during QMT practice prior to performing a step in the Quadrato Space.

A specific non-aerobic category of PA which has consistently been found to promote neuroplasticity and improve EFs is Mindful Movement (MM) [46]. Examples of MM practice include Hata Yoga, Tai Chi, and Aikido. While they differ from one another, all are thought to be characterized by focus of attention and awareness on body movement in the present moment, excluding all the other possible thoughts, and by typical flowing body movements that can range from high-level dynamic movements to static postures. A relatively new non-aerobic, coordination-demanding form of PA is the Quadrato Motor Training (QMT), developed by Patrizio Paoletti (see [47] for a review). QMT requires balance, coordinative movements, increased awareness to the body and its location in space, and enhanced divided attention to motor responses and cognitive processing [47]. QMT requires participants to stand in one corner of a 50 × 50 cm square, the Quadrato space, and produce the correct direction of movement into it, as indicated by a specific sequence of verbal instructions [47]. At each corner of the Quadrato space, labeled with numbers from 1 to 4, there are three possible directions to move in: right or left; forward or backward; and diagonally. Moreover, based on the specific instruction, there can be a fourth option that requires movement to be withheld (e.g., when the verbal instruction indicates movement from corner 4 to corner 4). Thus, training comprises 12 possible movements plus one non-movement option (See Figure 1). Participants are instructed to keep their eyes focused straight ahead without fixating on any specific point, with their hands by their sides, and to continue with the next instruction and not stop after making a mistake.

## 2. QMT Effects: Neuropsychological Research

**Figure 2:**
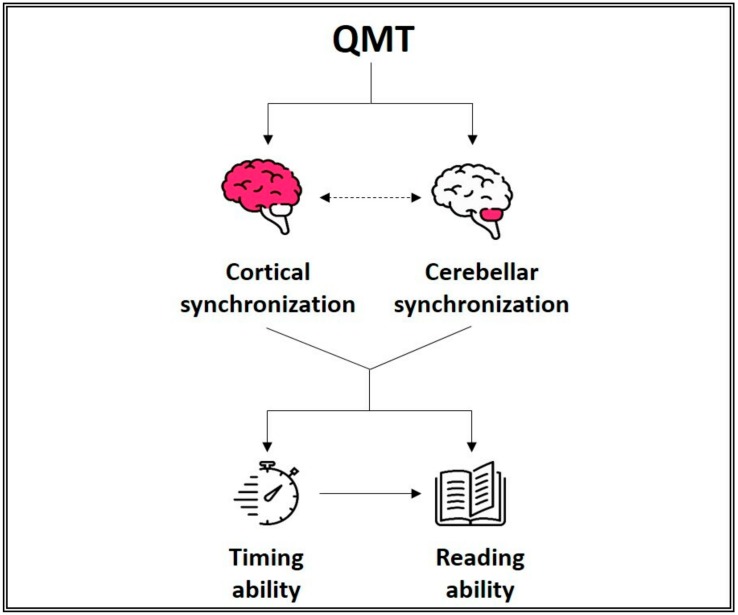
Interconnected relationship between movement and cognitive function. The relationship is mediated via two main interrelated routes: (1) slow rhythm oscillations and functional connectivity; (2) molecular effects and structural changes. Adapted from [34].

In the past decade, several studies have investigated the effects of QMT on brain structure as well as on cognitive and psycho-emotional functions. Studies published before 2015 were included in a previous review, in which a theoretical model was proposed. The model is based on a multimodal approach, uniting QMT-induced electrophysiological, neuroanatomical, and molecular changes, and suggests that changes in cerebellar slow rhythm oscillations are one of the main mechanisms mediating between QMT and improved cognitive functions (Figure 2) [47]. In the last four years, additional electrophysiological, neuroanatomical, and behavioral studies were conducted to examine the effects of QMT on psycho-emotional well-being, and elucidate their underlying mechanisms. The primary aim of the present overview is to summarize QMT-induced effects and associated neurobiological mechanisms that enable an internal environment of improved cognition and psycho-emotional well-being. This allows us to extend Paoletti’s neuro-psycho-educational model [48], which was then also detailed by Pesce and Ben-Soussan [29], in a way which could be relevant for MM practices and PA in general. Finally, we will address the broader implications of improving cognitive and psycho-emotional well-being through QMT for environmental research and public health.

### 2.1. Electrophysiological Effects of QMT

In the field of electrophysiological research, the most common measure used to study neural synchronization are EEG power, which reflects synchronization of the activity of thousands to millions of cortical neurons in the same neuronal population, and various measures of functional connectivity. One such measure is coherence, which reflects synchronization of the activity of two distinct neuronal populations that can be located in the same hemisphere (intra-hemispheric) or in different hemispheres (inter-hemispheric).

Previous studies have shown that different forms of PA, including mind-body training [49,50,51,52,53,54,55] as well as mindfulness practices [56,57,58,59], can modulate electrical brain activity. To uncover the potential underlying electrophysiological mechanisms of QMT-induced cognitive changes, EEG power and coherence were first examined. Studies on healthy populations demonstrated that QMT promotes EEG power [59,60] and coherence [34,61,62,63], especially in the theta (6.5–8 Hz) and alpha (8–12 Hz) band.

Though the functional significance of alpha is still under debate, decreases in alpha power are believed to reflect enhanced externally-oriented attention, while increases indicate that attention is focused on the inner environment [64]. Thus, QMT seems to promote internally oriented attention. Not surprisingly, QMT has also been found to increase reflectivity [34], mindfulness, and altered states of consciousness [65], as discussed below (Section 2.4).

Both single sessions and protracted periods of QMT resulted in increased intra- and inter-hemispheric functional connectivity in the theta and alpha bands [34,61,62,63]. Increased theta and alpha functional connectivity is thought to reflect improved cognitive functions and higher states of consciousness, due to better integration of information and communication across brain regions [66,67,68], such that these results support QMT’s capacity to promote cognitive and psycho-emotional well-being (see Section 2.4).

QMT has also been studied in neurodevelopmental and neurodegenerative disorders [59,69]. Dyslexic and normal readers were compared in a MEG-based study investigating potential QMT-related modulation of both cortical and cerebellar alpha power and coherence [59]. The results showed reduced cerebellar alpha power in the dyslexic group compared to normal readers at baseline and increased cerebellar alpha power in the dyslexic group upon completion of training. Previous studies suggested that the cerebellum is a potential biomarker of dyslexia, which can involve both phonological difficulties and sensorimotor deficits [70,71,72,73]. In addition, the cerebellum plays a fundamental role in motor functioning as well as cognition [74]. In particular, timing ability [75], EFs [4], and language [76] are often found to be deficient in dyslexia e.g., [70,77]. As such, convergent evidence suggests that QMT could be beneficial in improving the motor and cognitive symptoms of dyslexia.

QMT-induced electrophysiological effects were also studied in relation to mild cognitive impairment (MCI) [69], as MCI patients are known to demonstrate decreased alpha power [78,79,80,81,82,83] and coherence [84,85,86,87]. In a study on amnesic senior adults with MCI, a daily 4-week QMT program was compared with simple walking motor training (WMT). Significant enhancement in right occipital-parietal and occipital-temporal alpha was found in the QMT group, in contrast to the control group. Notably, prior to training, the right alpha functional connectivity between occipito-parietal and occipito-temporal areas was lower in the MCI group than in normative seniors. Furthermore, MCI participants who followed WMT showed a decrease in power alpha/delta ration, while those who followed QMT remained stable [85]. Although future studies should be conducted on the subject, the current results suggest that QMT may induce beneficial enhancement of the EEG markers that are typically damaged in individuals with MCI. It is also noteworthy that the changes in EEG functional connectivity were very similar to those found by Lasaponara and colleagues [61] in a healthy population.

Taken together, these studies demonstrate that QMT is an MM practice that can improve brain functioning and communication in both healthy and clinical populations. Since functional changes may also be related to structural changes, the next section addresses the effects of QMT on gray and white matter.

### 2.2. Neuroanatomical Effects of QMT

Structural imaging techniques allow researchers to differentiate between the two main central nervous system tissues, grey matter (GM) and white matter (WM). Fractional anisotropy (FA), axial diffusivity (AD), and radial diffusivity (RD) are considered primary markers of WM integrity [88,89,90,91]. In particular, increases in FA are thought to result from increased myelination, a higher number and greater size of axons, and better cell membrane properties [89,92]. In contrast, increases in AD and RD are thought to reflect decrements in axon density or caliber [93] and decreased myelination [94,95], respectively. Independent of the specific WM marker, increases in WM integrity have been associated with improved functional connectivity, cognitive and metacognitive functions, and motor performance [89]. Moreover, different studies have shown that people who are highly skilled in particular motor-cognitive disciplines have higher WM integrity than do non-skilled or non-expert individuals [89]. Similarly, people who followed either physical or meditative trainings showed increments in WM integrity [89,96,97,98,99,100,101,102,103,104,105]. Deterioration in WM integrity, on the other hand, is linked to the aging process and to cognitive decline, psychiatric disorders, and neurological diseases [106,107,108].

The three studies that investigated QMT-related structural changes in the brain [109,110,111] indicated increased GM and WM in brain areas that are mainly involved in sensorimotor, cognitive, and emotional control. Specifically, the studies that focused on GM changes after QMT predominantly found increases in the cerebellum and frontal lobe [109,110]. Though the cerebellum is known to be an especially important brain structure for sensory and motor functioning, it is also highly involved in cognitive functioning especially through the projections to and from the frontal lobe [4,112,113]. These two brain structures are strictly interrelated, and neuroimaging studies have found that activation in the cerebellum is closely coupled with frontal cortex activation [4,112,113]. For example, cerebellar activation increases when a cognitive task is difficult as compared to easier tasks, when it is new as compared to familiar tasks, and when it requires a high level of attention and concentration as compared to tasks requiring low cognitive effort [4]. Meanwhile, frontal cortex activation increases when a motor task requires holding and/or working with information in mind, resisting distraction and staying on task, and inhibiting inappropriate behaviors that could compromise motor performance [4].

Studies addressing WM [109,110,111] showed that QMT increased FA and decreased RD in the cerebellum and, more specifically, in the cerebellar peduncles, a brain structure involved in the cerebro-cerebellar interaction and in the connection of this pathway with the midbrain [113]. QMT also resulted in enhanced FA and reduced RD in the anterior thalamic radiations, which are generally related to EFs, memory encoding, and planning of complex behaviors [114,115], gait stability, and speed [116,117]. Significant FA increases were also found in both the left and right uncinate fasciculi, which play an important role in emotion regulation, learning, and language functions [118,119]. Other significant FA increases were found in the body of the corpus callosum, suggesting an increase of inter-hemispheric communication between frontal areas [120]. Finally, QMT induced FA increments in sensorimotor tracts (e.g., corticospinal tract), and other brain tracts involved in verbal and visual memory, language, and attention, including the superior and inferior longitudinal and inferior fronto-occipital fasciculi. In fact, increased FA/decreased RD in the right anterior thalamic radiation and left superior longitudinal fasciculus were associated with training-induced improvements in originality and general self-efficacy (see Section 2.4).

In light of these results, it appears that QMT-induced neuroanatomical changes further support the electrophysiological changes described in the previous Section 2.1. Other researchers [31,32,35] have suggested that these QMT-induced functional and structural brain changes are mediated by neurotrophic factor level modifications. Therefore, in the next section, we will report on the molecular effects of QMT on neurotrophins.

### 2.3. Molecular Effects of QMT

Neurotrophins are proteins that have nourishing or sustaining effects on neurons [121]. The first neurotrophic factor discovered was the nerve growth factor (NGF) in work conducted by Rita Levi-Montalcini and colleagues [122]. In the following years, other neurotrophins were discovered, including brain-derived neurotrophic factor (BDNF) [123], the most abundant neurotrophin in the growth factor family [124]. Both NGF and BDNF are synthetized as pro-neurotrophins (i.e., as precursor forms), called pro-NGF and pro-BDNF, respectively, and are released in the synaptic space [121].

Knowledge of the biology of neurotrophins and the modification of their release in response to external environmental stimuli has exploded in the last two decades. It is evident that this family of growth factors plays a fundamental role in normal nervous system development and adult physiology, as well as in the pathophysiology of the brain [121]. Considering that they are involved in critical activity-dependent processes, like synapse development and plasticity, synaptic efficacy, neuronal development, connectivity, and survival [121], the scientific study of neurotrophins in both mature and precursor forms is fundamental to our understanding of cognitive functioning and emotional well-being and to changes in these factors in response to training.

Most of the studies related to PA-induced effects on human neurotrophic factor levels investigated changes in BDNF. To the best of our knowledge, QMT is the only form of PA that has been investigated with respect to changes in human NGF levels [38] and their association with BDNF [125]. More specifically, three studies explored QMT-induced changes in neurotrophins. This is a novelty in the field of PA and, in general, in the field of molecular biology, since only one previous correlational study as far as we know has investigated the reciprocal behavior of NGF and BDNF was conducted during development [126].

With respect to proNGF, researchers found a decrease in healthy adults and children following 4 weeks of daily QMT practice, compared to a control group who underwent a 4-week daily walking training program [38]. The opposite results were found when participants underwent a 12-week daily QMT program, who showed an increase in proNGF in comparison with a control group who underwent 12 weeks of daily walking training [125]. A possible explanation for these apparently contradictory results might be that proNGF decreases after 4 weeks of QMT due to its fast utilization, which is related to enhanced neuroplasticity. Subsequently, this proNGF consumption stimulates further re-synthesis in the following 8 weeks, leading to an increase in proNGF [125]. In other words, the change in the neurotrophic level may depend on the length of training. This explanation may help resolve contradictions in the literature on PA-induced neurotrophic changes, which show the same trend e.g., [127,128,129]. A similar explanation has been proposed by Babaei and colleagues (2014) [130], who hypothesized that prolonged PA may improve the neurotrophic uptake mechanisms into the CNS, because of increased receptor sensitivity in the brain, which, in turn, increases brain volume and decreases the peripheral neurotrophic concentration.

With respect to proBDNF, a previous study found an increase after 12 weeks of daily QMT practice, which was associated with enhanced GM and WM in the cerebellum [110]. Although preliminary, this study not only improved our understanding of QMT effects at the molecular level but also linked QMT-induced neuroanatomical changes to variations in neurotrophic level. A trend toward proBDNF increase was also observed in participants who underwent 12 weeks of daily QMT, in comparison to a walking training control group [125]. Notably, this change in proBDNF was positively associated with the increase in proNGF in participants who practiced QMT [125].

As asserted above, increased release of neurotrophic factors is thought to be one of the main mediation mechanisms by which PA induces neuroplasticity [31,32,35]. This, in turn, activates a series of structural and functional brain changes like those mentioned above. In the following section, we will discuss the behavioral effects of QMT and their association with electrophysiological, neuroanatomical, and molecular changes.

### 2.4. Behavioral Effects of QMT and Associations with Other Parameters

In light of the results reported in the previous sections, it can be argued that such structural and functional brain changes as well as molecular changes could predict actual behavioral improvements in cognitive and/or emotional domains. However, as recently pointed out by Diamond and Ling [46], there are cases in which changes in brain structure and/or function do not lead to improvements in cognition and emotional regulation, for instance, because of failure to reach a critical threshold. As such, studying the direct effects of QMT at the cognitive and psycho-emotional level was considered extremely important, and several studies explored QMT-induced behavioral changes related to both cognition [38,59,62,63,65,109,111,131,132] and emotion [65,111,133].

One of the cognitive functions that has been studied most in relation to QMT is divergent thinking [38,62,109,111], which is defined as the ability to generate multiple ideas in response to given open-ended problems [134] and considered a useful estimate of creativity [135,136]. Studies showed an improvement in divergent thinking after a single session of QMT [61] and after a protracted period of practice [38,111]. These changes were associated with increased frontal alpha coherence [62], changes in WM in the right anterior thalamic radiation and left superior longitudinal fasciculus [111], and changes in cerebellar GM and WM [109]. Increased creative thinking was also associated with QMT-induced changes in proNGF in children and adolescents [38]. These results support the possibility that QMT induces structural and functional changes in brain areas that are related to creativity. Indeed, as suggested by several researchers, creativity is a high cognitive function that requires widespread brain activation [134,135,137], with connections between the frontal cortex and the cerebellum playing a central role [4,137].

Reaction time was also positively influenced by QMT [38,62]. Healthy adults who underwent a single session of QMT showed faster reaction times, which were associated with increased frontal alpha power [62], supporting the role of frontal alpha activity in improved cognition and the capacity of QMT to promote it.

Researchers have also investigated the acute effects of QMT on spatial cognition and reflectivity [34], or the capacity to exercise introspection by examining conscious thoughts and feelings, resulting in the inhibition of habitual thoughts or behaviors. Reflectivity can be measured directly by a spatial task called the Hidden Figures Tests, which assesses field dependence-independence [138]. Using this task, Ben-Soussan et al. [34] showed improved spatial cognition and reflectivity in groups who underwent a single session of QMT, in comparison to two control groups that underwent either simple motor or verbal training. The improvements were thought to stem from changes in functional connectivity, as evidenced by changes in intra- and inter-hemispheric coherence in theta and alpha bands [34].

Timing is a crucial function involved in everyday activities such as speaking and reading. One way to measure timing ability is to generate a response when a given interval (in seconds) has elapsed, as in the commonly used Time Production task [139]. A cross-sectional study revealed that advanced QMT practitioners showed longer produced durations than did Aikido practitioners and a physically inactive control group [131]. These findings suggest that QMT has the capacity to dilate the subjective time experienced, possibly by inducing greater awareness of the present moment and of the body. In fact, the production of longer time durations can be explained generally by increased relaxation, decreased arousal, and increased size of subjective time units [140].

**Figure 3:**
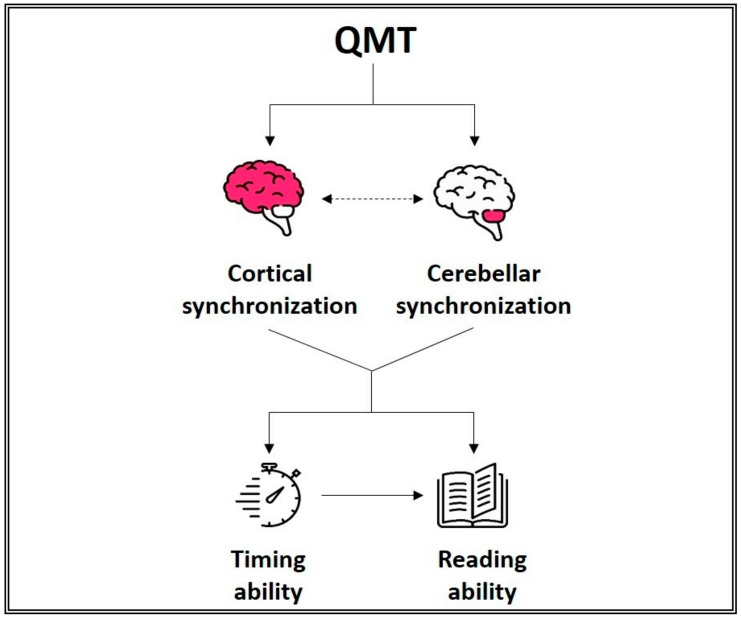
The potential mechanisms by which QMT improves reading ability. QMT increases cortico-cerebellar synchronization that, in turn, leads to both a direct improvement in reading ability and an indirect improvement in reading ability through enhanced timing ability (this indirect pathway still requires verification).

Since timing is one of the cognitive functions affected in dyslexia [77,101,141,142,143,144], as a first step towards examining timing in this population, researchers explored whether and how QMT influences the perception of time in dyslexic and normal readers. Results showed longer time durations in females with dyslexia after one month of intensive QMT [132]. Integrating findings from studies on typical readers [63,131] and individuals with dyslexia [59,132], the researchers hypothesized that better functioning of the cerebellum and frontal and temporal areas might mediate the QMT-induced dilation of time duration. In fact, QMT increased functional synchronization in the frontal and temporal areas, which was associated with a change in time production [63], and improved neural synchronization in the cerebellum [59]; these are brain areas that are highly involved in timing ability [75,145]. This hypothesis is further supported by the fact that QMT-induced improvements in cortical and cerebellar functioning among individuals with dyslexia were associated with improved reading performance [59]. It is known that reading strongly depends on timing ability [146]. Moreover, the cerebellum plays a fundamental role in language [76] through (among other things) its projections with frontal and prefrontal areas [4], and its dysfunction is considered a potential biomarker for dyslexia [70,71,72,73]. It can therefore be inferred that QMT enhances cortical and cerebellar functional synchronization in individuals with dyslexia, which leads to improved timing ability that, in turn, positively affects reading skills (Figure 3).

Through quantitative methodologies, such as psychometric tests, researchers in the field of cognitive and affective neuroscience can investigate the results of a given intervention. However, less is known about the processes by which interventions induce such effects. Often, qualitative methodologies such as semi-structured interviews can shed light on underlying processes. Since subjective experience is as important as psychometrically tested aspects of cognition, Ben-Soussan et al. [65] aimed to investigate QMT-induced cognitive and psycho-emotional experiences using a semi-structured oral interview following one of the following QMT interventions: 28 days of daily QMT for breathing meditation practitioners (M28) and healthy non-breathing meditation practitioners (C28); and a single session of QMT for other breathing meditation practitioners (M1). Three primary categories emerged from the interviews, which were called “Attentional Effort”, “Mindfulness”, and “Altered State of Consciousness” (ASC). Although the attention-related experience, which included attention and concentration, tiredness, and distraction, was present in all groups, it emerged more strongly in novices (C28) after protracted QMT practice. This suggests that non-practitioners invested more attentional effort in QMT practice than did meditators, as QMT may require greater effort until expertise is achieved. This explanation is in line with the idea that cognitive resources must be challenged continually, and not just used, in order to elicit improvements [45]. In both meditation groups (M28 and M1), experiences related to the Mindfulness category, which included *the ability to wait (to the next instruction)*, positive emotion, and stability and harmony of the body were most often reported, with significantly more experiences reported in the M28 group than in C28. This might reflect the mindfulness trait that often characterizes expert meditators, which allows them to reach deeper mindful states after practice [56]. Finally, participation in a 4-week QMT program increased ASC, that is, spontaneous visualization, intuition, and sense of wonder, among meditation practitioners (M28) as well as non-meditation practitioners (C28), in contrast to participants who received a single session of QMT (M1). This last finding emphasizes the importance of a protracted period of QMT practice in promoting and facilitating ASC experiences. This shift in the relationship between thoughts and feelings, which may then be observed as arising phenomena instead of occupying full attention, is common to many meditative practices [147]. Therefore, at the behavioral level, QMT not only induced cognitive improvements but also promoted psycho-emotional well-being.

The psycho-emotional benefits instigated by QMT have also been investigated using quantitative instruments. More specifically, two studies investigated the potential role of QMT in promoting psycho-emotional changes, particularly with respect to affect balance [133] and self-efficacy [111,133]. Affect balance, as assessed using the Bradburn’s Affect Balance Scale, can be defined as the difference between positive and negative affect, in which higher scores reflect greater prevalence of positive emotions [148]. Investigating whether a combination of QMT and breathing meditation influenced affect balance more than intense breathing meditation alone, researchers found increased affect balance in participants who underwent one week of intense QMT-breathing meditation practice [133]. These results suggest that it is possible to enhance positive emotion through QMT, which may in turn promote resilience and copying with stress [48,149,150]. They also emphasize the importance of introducing body movements into meditative practices.

Self-efficacy is also believed to constitute a buffer against stressful experiences, since highly self-efficacious individuals perceive demands as challenging instead of threatening [151]. A study investigating QMT effects on self-efficacy showed improvements after one week of intense QMT combined with breathing meditation, in comparison to a control group who underwent breathing meditation alone [133]. An association was also found between enhanced self-efficacy and increased WM integrity in the anterior thalamic radiations and left superior longitudinal fasciculus [111], brain areas found to be altered in anxious and depressed patients [152,153]. This was the first study to explore white matter correlates of training-induced changes in self-efficacy and, taken together, its results supported the importance of QMT in promoting cognitive as well as psycho-emotional well-being. In the following section, we will better explain the unique aspects that make QMT an external movement-based environment that elicits cognitive improvements and promotes psycho-emotional well-being, and what differentiates it from other forms of MM. We will also discuss the importance of introducing QMT practice in various health promoting, clinical, and educational settings.

## 3. What May Differentiate QMT from Other Forms of MM?

Most of the QMT studies described above compared QMT groups to control groups that isolated the motor component from the cognitive component of QMT. Training characterized by the same motor engagement but without cognitive effort was called Simple Motor Training (SMT), in which participants performed the same practice as QMT but without choice requirements. The cognitive training that was identical to QMT but without the motor component was called Verbal Training (VT), in which participants were instructed to respond verbally rather than with movements. These studies demonstrated greater improvements following QMT than after both VT and SMT [47], suggesting that the effects induced by QMT are due to intrinsic embodied cognition.

To understand what is special in QMT, we need to start from its basic instructions. As introduced in Section 1.2, QMT is based on a Quadrato space, which is a square divided into four corners, labeled with numbers from 1 to 4. Starting from the first corner, the participant is required to produce a step in response to a specific verbal instruction presented in an audio tape recording [47]. For example, an instruction can be “4–3”, which means that the participant should move from corner number 4 to corner number 3. To convert the verbal instruction to a correct movement into the Quadrato space, the participant must know where s/he is. This task requires the participant to hold and manipulate information, which is the role of working memory [45,154]. QMT is also characterized by a continued state of attention to and waiting for the next instruction. Thus, the participant must remain focused on the task over a relatively long period of time (possibly several minutes) and suspend the tendency toward habitual and instinctive movements. It is also important to consider that a possible instruction could be “4–4,” which requires inhibition of the impulse to move whenever the voice command is heard. These three QMT features mainly require sustained attention [45,154] and inhibitory control [45,154]. Furthermore, inhibitory control plays a role in continuing to the next instruction and not stopping when a mistake occurs. Attentional efforts must also be divided between body and space.

All the cognitive functions elicited by QMT promote increased presence, namely, a “here and now” condition, which is a basic characteristic of mindfulness-based practices, including MM [155]. Another characteristic that QMT has in common with other mindfulness-based practices involves being nonjudgmental [155,156]. QMT instructions are to not slow down or correct yourself when you make a mistake, to just keep going. In other words, to avoid feeling upset about the error. Not judging or berating oneself is a central aspect of most mindfulness practices. In particular, QMT incorporates the three independent phases of a mindful act [157]: (1) suspension from the habitual act of allowing the mind and the body to go where they want; (2) redirection of attention toward the external cue and the internally generated movement; and (3) receptivity toward the experience.

Importantly, we have used the term *Mindful Movement* to describe practices like Tai Chi, Aikido, and Hata Yoga, which previously have also been defined as “meditative movement” by Larkey and colleagues [155]. According to their definition, meditative movements are defined by several characteristics, including (1) a focus of the mind on the movement practice in the present moment, excluding all other thoughts; (2) inclusion of some form of body movement; (3) a more or less explicit focus on breathing; and (4) a deep state of relaxation as an intrinsic practice element [155]. While the first three components of Larkey et al.’s definition of meditative movement are also included in mindfulness-based practices, the fourth one (i.e., deep relaxation) is not always considered an integral part of mindfulness, independently of the used definition e.g., [157].

What, then, differentiates QMT from other forms of MM? First of all, QMT does not include a focus on breathing. However, the main difference is that during QMT, participants do not know the next movements that they will perform in advance, as in other MM practices such as Tai Chi, Qui Gong, and Aikido. Participants know what to expect but do not know exactly what movements they will be required to perform. In QMT, the precise timing of the movement is externally paced on a momentary basis, whereas in other MM, movement sequences are usually performed and internally passed once the instruction is given. In fact, the continual state of attending and waiting for the next instruction during QMT results in the participants being obliged to enter this state of suspending the tendency for habitual movement, that of moving where and when you want [59,131]. This state requires more sustained attention, working memory, and inhibitory control. Therefore, this intrinsic characteristic of QMT forces participants to stay ready to act and continuously divide attention between the verbal instructions in parallel to the position in space. The second important distinctive feature of QMT is its short and modifiable duration. This means that QMT is a MM method that can potentially be practiced every day, once or even more, as it does not need to much time. This is particularly important considering that perceived lack of time is one of the main reasons that people refrain from regular PA [158]. Moreover, QMT could eventually be practiced anywhere, requires limited space, and, after a few days of practice with a specialized trainer, can be performed without an expert to lead the practice. These are other fundamental distinctive features that enhance people’ opportunities to increase the time spent in PAs. Finally, from a motor perspective, it is a relatively simple MM and, thus, potentially practicable by almost anyone, from childhood to late adulthood, in both healthy and clinical conditions.

## 4. Conclusions and Implication for Environmental Research and Public Health

A person’s neurobiological and physiological mechanisms (i.e., internal environment) and the space in which the person moves, acts, and lives (i.e., external environment) are closely interrelated. This is the reason that QMT, which can be defined as an external movement-based environment, is hypothesized to stimulate neuro-promotion and prevent neurodegeneration, potentially enabling the creation of an internal environment of improved cognitive and emotional regulation and enhanced well-being.

QMT may potentially serve as a powerful, suitable, and relevant tool for training in classrooms as well as in the public health system, to support and strengthen children and adolescents during critical periods of development, promote cognitive and psycho-emotional well-being, enhance personal and social awareness and responsibility, and possibly prevent and treat neurodevelopmental and neurodegenerative diseases. For these reasons, we are currently working on introducing QMT into elementary and middle school curricula, with the aim of studying its effects on EFs and academic performance. As one reviewer of this paper has mentioned, age-dependent QMT effects could be addressed in future research, perhaps with a particular emphasis on length of practice and velocity. Another direction is related to cerebellar and motor deficits, which occur in additional developmental disorders, not only in dyslexia. For example, in autism spectrum disorder, in parallel to the cognitive deficits, children suffer from different sensorimotor deficits. Future studies should further examine QMT in additional clinical settings, especially related to MCI and Alzheimer’s disease, to better investigate the mechanisms by which QMT can slow down cognitive, emotional, and motor degeneration. We are currently working in these directions. Furthermore, as one reviewer has remarked, the use of QMT in patients could also be tested with Parkinson’s disease—in parallel with severe motor deficits, these patients often experience cognitive decline and decreased mental flexibility.

Additional studies should be conducted to better understand the role of movement in different mindfulness practices compared with aerobic forms of PA. A combined examination of the possible inter-dependent change in motion, emotional and cognitive functions as well as motor skill acquisition could be crucial [29,159]. In this regard, it will be important to compare individually performed trainings with group-settings, especially considering the role of social engagement in cognitive and emotional well-being [29,45].
